# Incidence of Respiratory Virus-Associated Pneumonia in Urban Poor Young Children of Dhaka, Bangladesh, 2009–2011

**DOI:** 10.1371/journal.pone.0032056

**Published:** 2012-02-22

**Authors:** Nusrat Homaira, Stephen P. Luby, William A. Petri, Raija Vainionpaa, Mustafizur Rahman, Kamal Hossain, Cynthia B. Snider, Mahmudur Rahman, A. S. M. Alamgir, Farzina Zesmin, Masud Alam, Emily S. Gurley, Rashid Uz Zaman, Tasnim Azim, Dean D. Erdman, Alicia M. Fry, Joseph Bresee, Marc-Alain Widdowson, Rashidul Haque, Eduardo Azziz-Baumgartner

**Affiliations:** 1 International Centre for Diarrhoeal Disease Research, Bangladesh (icddr,b), Dhaka, Bangladesh; 2 Centers for Disease Control and Prevention (CDC), Atlanta, Georgia, United States of America; 3 University of Virginia, Jack Jouett, Virginia, United States of America; 4 University of Turku, Turku, Finland; 5 Institute of Epidemiology Disease Control and Research, (IEDCR), Dhaka, Bangladesh; University of Witwatersrand, South Africa

## Abstract

**Background:**

Pneumonia is the leading cause of childhood death in Bangladesh. We conducted a longitudinal study to estimate the incidence of virus-associated pneumonia in children aged <2 years in a low-income urban community in Dhaka, Bangladesh.

**Methods:**

We followed a cohort of children for two years. We collected nasal washes when children presented with respiratory symptoms. Study physicians diagnosed children with cough and age-specific tachypnea and positive lung findings as pneumonia case-patients. We tested respiratory samples for respiratory syncytial virus (RSV), rhinoviruses, human metapneumovirus (HMPV), influenza viruses, human parainfluenza viruses (HPIV 1, 2, 3), and adenoviruses using real-time reverse transcription polymerase chain reaction assays.

**Results:**

Between April 2009–March 2011, we followed 515 children for 730 child-years. We identified a total of 378 pneumonia episodes, 77% of the episodes were associated with a respiratory viral pathogen. The overall incidence of pneumonia associated with a respiratory virus infection was 40/100 child-years. The annual incidence of pneumonia/100 child-years associated with a specific respiratory virus in children aged <2years was 12.5 for RSV, 6 for rhinoviruses, 6 for HMPV, 4 for influenza viruses, 3 for HPIV and 2 for adenoviruses.

**Conclusion:**

Young children in Dhaka are at high risk of childhood pneumonia and the majority of these episodes are associated with viral pathogens. Developing effective low-cost strategies for prevention are a high priority.

## Introduction

Pneumonia is one of the major causes of childhood death causing annually 2 million deaths worldwide [1], [2], [3], [4]. Approximately 68% of these deaths occur in the first year of life [5]. Viral pathogens, primarily respiratory syncytial virus (RSV), human parainfluenza viruses (HPIVs), human metapneumovirus (HMPV), influenza A and B viruses and adenoviruses [3], [6], [7], [8], [9], [10], [11] are major contributors to community-acquired pneumonia, especially in the first years of life [4]. There is a growing body of literature describing the etiology and incidence of pneumonia associated with different respiratory viral infections among hospitalized children but less information is available on the community-based incidence of virus specific pneumonia. The incidence of pneumonia is approximately 10 times higher in low-income countries than in high-income countries [1]. In low-income countries hospital-based incidence may underestimate the true burden of disease as many children with community-acquired pneumonia may never seek medical care due to limited access to health care services. Community-based incidence data may help better define risk groups and provide evidence that can be used to develop effective intervention strategies [12].

According to WHO estimates Bangladesh is one of the 10 countries where two thirds of the pneumonia death are concentrated [13]. A study conducted in Dhaka, the capital city of the country suggested that 22% of childhood deaths occurred from respiratory infection [14]. Approximately 14–20% of children aged <2 years with respiratory infection in Bangladesh never seek any health care services [15]. Furthermore Bangladesh has one of the highest population densities in the world, approximately 1000 people/km^2^ and has a prevalence of childhood malnutrition of 41% [16], [17]. Malnutrition, overcrowding and lack of access to health care may contribute to increased mortality and incidence of respiratory diseases, especially in the very young. There are limited data about the contribution of respiratory viruses to incidence of pneumonia or death in children <2 years in Bangladesh. We conducted a longitudinal study in a low income urban neighborhood of Dhaka, Bangladesh to estimate the community-based incidence of pneumonia associated with laboratory-confirmed respiratory virus infection among children aged <2 years.

## Methods

### Ethics Statement

We obtained written informed consent from the mother, father and an adult primary caregiver for all children enrolled in the study. The Institutional Review Board at International Centre for Diarrhoeal Disease Research, Bangladesh, (icddr,b) approved the study and the Institutional Review Board at Centers for Disease Control and Prevention (CDC), USA deferred to icddrb's approval.

### Study site

We conducted our study in ward three of Pallabi thana (“thana” is the lowest administrative unit in Bangladesh). This ward of low-income community is in the north-west part of Dhaka and covers an area of 3.4 sq. km. The estimated population of the ward is 69,960; half of its population is male. The mean literacy rate is 69% for males and 59% for females [18].

### The cohort

In January 2008, icddr,b initiated surveillance among a cohort of children in the study area to assess immunity to human amebiasis. All children who were born in the study area and lived within 1.5 kms of the study clinic, were enrolled at birth and followed prospectively. Our study was conducted on this existing birth cohort of children. In April 2009, when we started our longitudinal respiratory disease study we re-enrolled all children who were already participating in the amebiasis birth cohort and were aged 0–2 years. Subsequently we treated the population as an open cohort because we continued to enroll all children who were born in the study area after the April 2009 enrollment. We subsequently followed the cohort of children including children who were enrolled in the beginning of the study in April 2009 and children who were born after beginning of the study until March 2011. Children who were already part of the ongoing amebiasis birth cohort were not under any interventional study.

### Data collection

As part of on the ongoing amebiasis study, field assistants measured height and weight of each of the enrolled child within three days of birth. When we re-enrolled children into our study which began in April 2009, field workers trained in collecting surveillance data using hand held devices collected additional information from each child, including level of parental education and breast feeding practices. Prospectively, field workers visited each child at home twice weekly for two years of the study, to identify whether the child developed any major symptoms of respiratory illness, including subjective fever; rapid, laboured or noisy breathing; lethargy; cyanosis; inability to drink; or convulsion or minor symptomsincluding cough, rhinorrhoea, sore throat, muscle or joint pain, chills, headache, irritability, decreased activity and repeated vomiting within the last 72 hours of follow-up [19]. Field workers referred all children who developed at least one major symptom or two minor symptoms to the study clinic located within the study area.

In the clinic, study physicians collected a history of illness including signs and symptoms using a structured questionnaire, conducted a physical examination, and collected nasopharyngeal wash from each child every time a child presented with a new episode of reported or documented fever (T≥38°C) or respiratory symptoms including cough, nasal discharge or difficulty in breathing. An episode was considered to be new if the child had been symptom free in the preceding 7 days.

The study physician diagnosed a child with acute respiratory tract infection (ARI) if the child had cough and/or runny nose and diagnosed a child with pneumonia, if the child had cough or difficulty breathing with age-specific tachypnea using WHO classification [20] including the following respiratory signs: crepitation, ronchi and wheezing. In the study clinic all children received free clinical care using standard WHO recommendations [20] and also free referral services including hospitalization in tertiary hospitals when needed.

### Sample collection and laboratory analysis

Study physicians collected a nasopharyngeal wash sample by attaching a butterfly catheter (needle removed), to a 10 ml syringe containing 5 ml normal saline, placing the child in a 30° semi-Fowler position with the head slightly angled forward, inserting the catheter between 2 and 3 cm into the nares, injecting the saline, and immediately applying suction while removing the catheter [19]. The wash sample was then placed in viral transport medium (containing Dulbecco Modified Eagle Medium), and stored at 4°C in the study clinic refrigerator. At the end of each day, within eight hours of sample collection, a field assistant transported all respiratory samples to icddr,b's virology laboratory using cold box. At the virology laboratory the samples were stored at or below −70°C temperature until analyzed. Virologists at icddr,b performed real-time reverse transcription polymerase chain reaction (rRT-PCR) assays to detect influenza A and B viruses, RSV, adenoviruses, HPIVs 1, 2 and 3, human rhinoviruses, and HMPV using primers and probes supplied by the Centers for Disease Control and Prevention (CDC), Atlanta [primer/probe sequences and assay protocols for non-influenza respiratory viruses available from CDC on request].

### Data analyses

We observed each child from the start of enrollment of the child in the study and continued until the end of the follow-up period (March 2011), until the child left the study, or the family of the child moved out of the study area to reside in some other area. We calculated incidence as the number of new events divided by child-years at risk. For calculating virus-specific incidences of ARI and pneumonia we restricted our analyses to children who were aged <2 years during the follow-up period and divided the child-years in to three age groups (0–6months, 6–12 months and 12–24 months). We used Poisson estimation to calculate incidence and 95% confidence interval around incidences. Using the date of illness onset, and the proportions of viral pathogen detected in the respiratory samples, we plotted timing of viral pneumonia in a histogram.

## Results

### Demographic characteristics

Our study began in April 2009 and ended in March 2011. We followed 515 children for 730 child-years of observation (median time each child was observed was 1.7 years); 283 (55%) of the children were male. Fifty two (10%) of the 515 children did not complete the study because they migrated out of the study area. Among 515 children, 263(51%) were born after the study began in April 2009. The median age of the cohort at enrollment in to our study was 0.5 months (inter-quartile range [IQR] 0.1–8 months). The median birth weight of the cohort children was 2663 gms (a birth weight of 2500 gm is considered to be standard birth weight) [21] (Table 1).

**Table 1:** Baseline information of a cohort children (n = 515) in urban low income neighborhood of Dhaka, Bangladesh during April 2009–March 2011.

Sex of the child (male), n (%)	283 (55%)
Median birth weight of birth cohort child, grams (IQR)	2663 gm (IQR 2343–2968 gm)
Children with weight for age Z-score (WAZ) <−2, n (%)	137 (27%)
Children with height for age Z-score (HAZ) <−2, n (%)	86 (17%)
Median duration of exclusive breast feeding in months, (IQR)	4 months (2–6 months)
Children with mothers without formal education, n (%)	194 (39%)
Children with fathers without formal education, n (%)	185 (37%)

### Clinical profile and laboratory diagnosis

Among the 515 children followed, 423 (82%) developed 1,322 episodes of respiratory infections during April 2009–March 2011. A total of 918 (69%) of the episodes were classified as ARI and 378 (29%) were diagnosed as pneumonia by the study physician. The remaining 26 (2%) episodes were diagnosed as otitis media or only cough by the study physician. Out of the 223 children who developed at least one episode of pneumonia, 47 (21%) developed two episodes and 22 (10%) developed three episodes. The main presenting symptoms among children diagnosed with pneumonia were cough (100%), tachypnea (100%), reported fever (98%) (53% of the pneumonia episodes also had documented fever), runny nose (97%), difficulty breathing (84%), crepitations (44%) and ronchi (33%). The study physician referred one child (0.3%) with pneumonia to a tertiary children hospital who was hospitalized due to aggravating symptoms even after treatment as per WHO guidelines. There was no other hospitalization associated with ARI or pneumonia and we did not observe any ARI or pneumonia related deaths during the study period.

We identified viral respiratory pathogens in 66% (610 of 918) of ARI episodes and in 77% (293 of 378) of pneumonia episodes (Table 2). The median age of children who developed an episode of ARI associated with detection of a respiratory virus was 9.5 months (IQR 6–16 months) and that of children who developed an episode of pneumonia associated with detection of a respiratory virus was eight months (IQR 5–14 months).

**Table 2:** Frequency of respiratory viral pathogens in episodes of respiratory symptoms (N = 1,322) in a low income urban neighborhood, Dhaka, Bangladesh (April 2009–March 2010).

Viral pathogen	ARI(N = 918)n (%)	Pneumonia(N = 378)n (%)
Respiratory syncytial virus	91(10)	85 (22)
Rhinoviruses	119(13)	46 (12)
Human metapneumovirus	48(5)	41 (11)
Influenza A/H3	40 (4)	17 (4.5)
2009 pandemic influenza A (H1N1) virus	35(4)	6 (2)
Influenza B	45(5)	3(1)
Human parainfluenza virus 1	25(3)	6 (2)
Human parainfluenza virus 3	46(5)	23 (6)
Adenoviruses	68(7)	14 (4)
Human parainfluenza virus 2	11(1)	4 (1)
Multiple viruses	81(9)	49 (13)
No virus identified	308 (34)	85 (22.5)

We also identified 41 cases of 2009 pandemic influenza A (2009 H1N1) virus infection among the cohort children. The first child with 2009 H1N1 infection was identified on 12 July, 2009, less than four weeks after the detection of the first 2009 H1N1 case on 19 June 2009 in Bangladesh [22]. Only six (15%) of the 41 children with 2009 H1N1 infection developed pneumonia. The median age at infection of children with 2009 H1N1 infection was 13 months (IQR 5.5–20 months); 67% were male.

### Incidence of pneumonia associated with respiratory virus infection

**Figure 1:**
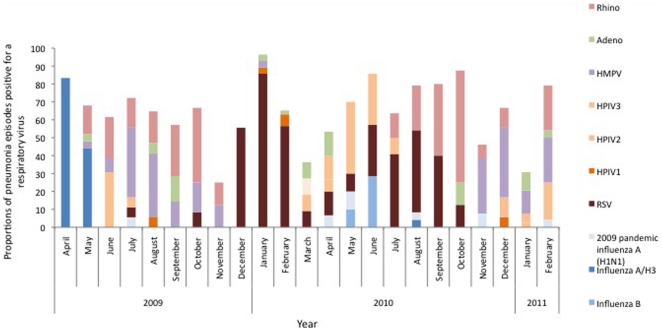
Monthly distribution of respiratory virus pathogens associated pneumonia in a cohort children in a low income urban neighborhood, Dhaka, Bangladesh (April 2009–March 2011).

The incidence of ARI was 125/100 child-years (95% CI 120–134) and pneumonia was 52/100 child-years (95% CI 47–57) in this cohort of children. The incidence of ARI and pneumonia associated with laboratory-confirmed respiratory virus infection was 80/100 child-years (95% CI 77–90) and 40/100 child-years (95% CI 36–45). Children aged 0–6 months were followed for 140 child-years at risk, children aged 6–12 months for 167 child-years and children aged 12–24 months for 297 child-years. The age-specific annual incidences of pneumonia/100 child-years associated with laboratory-confirmed respiratory virus infection were 32 (95% CI 21–39) for children aged 0–6 months, 51 (95% CI 41–63) for children aged 6–12 months and 44 (95% CI 37–52) for children aged 12–24 months. The annual incidence of pneumonia/100 child-years associated with specific respiratory viruses was 12.5 for RSV, 6 for rhinoviruses, 6 for HMPV, 4 for influenza viruses, 3 for HPIV 3 and 2 for adenoviruses (Table 3). Pneumonia associated with a respiratory virus infection was present throughout the year (Figure 1).

**Table 3:** Incidence of ARI and pneumonia per 100 child-years associated with different respiratory viral pathogens in a cohort of children aged <2 years in a low income urban neighborhood, Dhaka, Bangladesh (April 2009–March 2011).

	ARI/100 child-years (95% CI)				Pneumonia/100 child-years (95% CI)			
Virus etiology	0–6months	6–12months	12–24months	All age groups	0–6months	6–12months	12–24months	All age groups
RSV	8(4–14)	18(13–26)	13(10–18)	13(11–17)	15(10–23)	11(7–17)	12(9–17)	12.5(10–16)
Rhinoviruses	12(7–19)	27(20–36)	12(9–17)	17(14–20)	3(1–8)	8(4–13)	7(5–11)	6(5–9)
HMPV	4(1–9)	7(4–13)	9(6–13)	7(5–10)	1(0.5–6)	8(5–14)	6(4–10)	6(4–8)
Influenza	6(3–12)	20(14–28)	16(12–21)	15(12–18)	3(1–8)	5(3–10)	4(2–7)	4(3–6)
HPIV	1(0.4–6)	1(0.08–4)	7(4–10)	6(4–8)	1(0.1–5)	1(0.3–5)	1(0.3–3)	3(2–5)
Adenoviruses	1(0.1–5)	13(8–19)	12.5(9–17)	10(8–13)	0 (0.0–4)	2(1–6)	3(2–6)	2(1–3.5)

## Discussion

Our study suggests that childhood pneumonia is prevalent and viruses, especially RSV, are detected in the majority of urban poor children aged <2 years with pneumonia in Bangladesh. A total of 77% of all the episodes of pneumonia in this cohort had a respiratory virus detected which is similar to findings from other studies of young children where etiological agents of lower respiratory tract infections have been thoroughly investigated [23], [24], [25] confirming that respiratory viral pathogens are often associated with pneumonia in children <2 years of age [9], [26], [27]. Most of the published literature from low and high income countries describe incidence of respiratory viral pathogen associated pneumonia among hospitalized children, including those from Sub-Saharan Africa where pneumonia is one of the leading causes of childhood death [28], [29], [30], [31], [32]. Moreover, studies describing population based estimates have not included all the relevant respiratory viruses [19], [29], [30], [33], [34]. So the findings from our study provide more comprehensive data on virus etiologies and community based incidence of pneumonia in very young children.

Similar to findings from other studies, RSV was the most frequently identified virus in this cohort of children with pneumonia [9], [29], [35]. RSV infection in infants is frequently symptomatic, resulting in pneumonia and bronchiolitis in 30–70% of the cases [36]. Our rate of RSV associated pneumonia of 12.5/100 child-year was comparable to incidence of RSV infection of 12/100 child-years and 15/100 child-years in children <1 years of age with pneumonia symptoms in rural Nigeria and Kenya [28], [37]. Furthermore the incidence of RSV associated pneumonia was highest among children aged less than 6 months. Similar to findings from our study, RSV has been associated with pneumonia and severe lower respiratory illness compared to other respiratory viral and bacterial pathogens in children from other low and middle income countries including Thailand, Indonesia, Gambia and Kenya [29], [30], [38], [39]. These lines of evidence suggest that for children <2 years RSV may be one of the major contributors of respiratory illness in both low and middle-income countries.

Influenza viruses and HMPV were also individually detected in 7% and 9% of episodes of pneumonia. Influenza virus infections were associated with 10% of childhood pneumonia among children aged <5 years in a study conducted in another urban setting of Bangladesh [19]. Influenza virus infection in children may increase the risk of subsequent pneumococcal infection [3], [40], [41]. HMPV is also associated with lower respiratory tract infection in previously healthy children [42]. In a study conducted in Alaska, HMPV was more frequently identified in children <3 years of age hospitalized with respiratory infection than in children who did not develop respiratory symptoms suggesting that infection with the virus may be associated with severe illness [25]. We also detected rhinoviruses in more than 10% of the pneumonia episodes. However, rhinoviruses can be detected in asymptomatic children and can be shed in nasal mucosa for several weeks after illness onset. Therefore we cannot confirm the association of the virus with illness in the cohort children [25], [43].

In our study we observed increase in influenza activity in April–June of 2009 and 2010 which coincides with the influenza seasonality previously described in Bangladesh [44]. However as the study was conducted only for two years and due to lack of nationally representative data on timing of increase activity of respiratory viruses other than influenza we could not establish seasonality for other viruses.

Our study has several potential limitations. Our study population was drawn from a single urban community therefore the data may not be generalizable to the whole country. Nevertheless our incidence of pneumonia in this cohort of children was comparable to other studies done in other low-income settings including urban Bangladesh [1], [19]. We only followed 515 children limiting our capacity to document the effect of respiratory viruses associated ARI and pneumonia on mortality. Our diagnosis of clinical pneumonia was not confirmed by chest radiographic examination and it is possible that children with bronchiolitis were diagnosed as pneumonia case-patients. In a resource poor setting it might not be feasible to confirm each episodes of clinical pneumonia by radiographic investigation and WHO definition of pneumonia is widely used in community setting to diagnose pneumonia. We also did not evaluate for bacterial etiologies. However the objective of the study was to identity the viral pathogens associated with respiratory disease in children and literature suggests that viral pathogen are predominant contributors of community acquired childhood pneumonia [27].We did not collect respiratory specimens from children without respiratory symptoms for comparison, limiting our confidence in implicating all of the identified viruses as causative agents of the respiratory illnesses. There is evidence that rhinoviruses and adenoviruses can be detected in asymptomatic children or shed for long periods of time after infection [25], [43]. Nevertheless, most of the viruses tested for, including RSV, HPIVs, HMPV and influenza viruses are seldom identified in asymptomatic controls [25], [42], [45].

The study findings demonstrated a high incidence of pneumonia associated with respiratory virus infection among children aged <2 years. These children were under close follow up and received timely treatment and referral services when necessary. Children under such close surveillance are likely to under represent severe illness [46]. Other than influenza, there are no safe and effective vaccines to prevent childhood virus respiratory infections. Our data suggests that influenza infection was prevalent in children aged <6 months old, a group that could benefit from maternal immunization against influenza infection [47]. Further research on developing safe and effective vaccines, especially for RSV, could also play an instrumental role in reducing the disease burden. In the meantime, in low-income settings research focused on developing cost-effective interventions to address modifiable risk factors such as improved hygienic behavior, air quality, breast feeding practices and nutrition [2], [48], [49], [50] may help reduce the overall burden of respiratory tract infection in children.
